# A spread spectrum approach to time-domain near-infrared diffuse optical imaging using inexpensive optical transceiver modules

**DOI:** 10.1364/BOE.9.002648

**Published:** 2018-05-10

**Authors:** Konstantinos I. Papadimitriou, Laura A. Dempsey, Jeremy C. Hebden, Simon R. Arridge, Samuel Powell

**Affiliations:** 1 Department of Computer Science, University College London, WC1E 6BT, London, UK; 2 Department of Medical Physics and Biomedical Engineering, University College London, WC1E 6BT, London, UK

**Keywords:** (170.0110) Imaging systems, (170.3880) Medical and biological imaging, (170.4580) Optical diagnostics for medicine, (170.6920) Time-resolved imaging, (250.3140) Integrated optoelectronic circuits

## Abstract

We introduce a compact time-domain system for near-infrared spectroscopy using a spread spectrum technique. The proof-of-concept single channel instrument utilises a low-cost commercially available optical transceiver module as a light source, controlled by a Kintex 7 field programmable gate array (FPGA). The FPGA modulates the optical transceiver with maximum-length sequences at line rates up to 10Gb/s, allowing us to achieve an instrument response function with full width at half maximum under 600ps. The instrument is characterised through a set of detailed phantom measurements as well as proof-of-concept *in vivo* measurements, demonstrating performance comparable with conventional pulsed time-domain near-infrared spectroscopy systems.

## 1. Introduction

Over the last few decades, near-infrared (NIR) spectroscopy (NIRS) systems have significantly evolved from merely experimental tools to proper, non-invasive monitoring methods with numerous clinical applications [1,2]. The predominant application of NIRS is monitoring and evaluating increases or decreases in oxygenated hemoglobin (HbO_2_), de-oxygenated hemoglobin (HHb), total hemoglobin (tHb) and oxygen saturation (SO_2_) in tissues. The use of NIRS for the purpose of real-time brain haemodynamics recordings while the subject is performing a functional task is called functional NIRS (fNIRS) and is nowadays a popular technique for functional neuroimaging [3–7]. Moreover, the application of the NIRS principle to non-invasive tomographic and topographic imaging of tissues and organs has seen a rapid increase over this period, resulting in the generation of various diffuse optical topography and tomography (DOT) systems, which allow the two- and three dimensional reconstruction of tissues and organs by solving an inverse problem [8] to reconstruct images from measured raw data [9].

NIRS measurements are categorised into three different interrogation techniques: (i) continuous-wave (CW); (ii) frequency-domain (FD); (iii) time-domain (TD). Each one relies upon a different light emission and detection method, with its own advantages and disadvantages. CW NIRS instruments are capable of measuring only the intensity of the diffuse light, and consequently light scattering and absorption effects can not be easily differentiated. Despite this limitation, CW instruments are the most commercially exploited to date, in part owing to their simplicity and cost-effectiveness. On the other hand, within the last decade, the interest in FD and TD NIRS has started to steadily increase, mainly due to the fact that the richer set of measurements allow the recovery of both absorption and scattering information, but also to potentially superior contrast-to-noise properties and the ability to detect signals deeper within a turbid medium [10–14]. However, with FD NIRS depth discrimination can sometimes become challenging (e.g. in reflectance geometry measurements) in comparison with TD NIRS, which was proven to be the most efficient approach when it comes to depth sensitivity, recovery of the absolute value of the optical properties of the subjects under test and tomographic results [9].

As it has been repeatedly shown in literature [15], conventional TD techniques are challenging mainly due to their long integration time, their sensitivity to the ambient environment, especially in the case of time correlated single photon counting (TCSPC)-based detection, and due to the fact that the single light source often needs to be split and routed to the various sources. Classical TD NIRS instruments are bulky, expensive, typically employ sensitive optoelectronics, which are susceptible to vibrations, and switching between wavelengths could potentially be slow depending on the system’s architecture (in [15] Torricelli *et al.* report ∼10sec switching time for solid state lasers). In addition, it is worth mentioning that in the case where pulsed diode lasers are selected as light sources, the *warm-up* time required to achieve pulse time stability in the picosecond range may be long (potentially ≥60 min) [12, 15]. These factors limit the applicability of the technique to use in a hospital or research environment. A smaller, more robust implementation could facilitate wider applications in emergency medicine, enabling, for example, deployment in an ambulance.

In this work, we demonstrate experimental measured results from an alternative TD NIRS instrumentation setup, relying upon the spread spectrum method for time-of-flight (TOF) resolved measurements. The instrument’s light source utilises a commercially available, low-cost optical transceiver module, widely used in telecommunication applications, controlled by a Kintex 7 FPGA. The proposed setup can generate sub-ns system’s instrument response functions (IRFs), which are competitive with conventional pulsed excitation systems, exhibits sufficient accuracy and low noise properties, requires very short *warm-up* time and even in its current, proof-of-concept form occupies significantly less space compared to most traditional TD NIRS instruments. The paper is structured as follows: in Section 2, the implemented technique and instrument are demonstrated, while in Section 3, various characterisation experimental results are provided. In Section 4, tissue-equivalent phantom results are illustrated accompanied by a proof-of-concept *in vivo* arterial cuff occlusion experiment. Finally, a detailed discussion of the potentials, limitations and future improvements of the proposed setup is offered.

## 2. Excitation technique and system description

### 2.1. Spread spectrum technique

Typically in conventional single channel TD NIRS, an optical source and detector are placed appropriately around the object of interest. Subsequently, an ultra-short light pulse (circa few picoseconds pulse width) from the source is injected into a turbid medium, whilst the temporal point spread function (TPSF), i.e. the photon distribution of the time-of-flight (DTOF) is detected at the detector. A TPSF represents the tissue’s impulse response function which is the optimal measurement to characterise a system and is assessed based on the level of its delay, broadening or attenuation. A TPSF can be evaluated by a series of techniques for modelling and data analysis, such as (a) the forward model [16], (b) the inverse model [8] or (c) semi-empirical approaches [17]. In conventional TD NIRS instruments using pulse excitation (PE) for TCSPC, the following linear relationship holds between the measured TPSF and the real TPSF:

(1) $${\text{TPSF}}_{\text{Meas}.}^{\text{PE}}(t)={\text{IRF}}^{\text{PE}}(t)*{\text{TPSF}}_{\text{Real}}(t),$$

where * denotes convolution. With respect to the system’s IRF^PE^(t), the following relationship holds:

(2) $${\text{IRF}}^{\text{PE}}(t)=\text{Laser Pulse}(t)*\text{Source IRF}(t)*\text{Detector IRF}(t),$$

with the source and detector IRFs depending on the selected optical fibres (their length and dispersion affect the delay and the width of the measured TPSF, respectively), coupling between different optical components in the setup and between the optics and the subject under test [18]. From equations (1) and (2) it is implied that in order to obtain the true DTOF, deconvolution between the measured TPSF and the system’s IRF needs to be performed.

Recently spread spectrum methods were applied to TD NIRS as an alternative to pulsed excitation, which could help to overcome some of the instrumentational challenges discussed earlier. Spread spectrum techniques have been used for long time in telecommunication applications and their main advantages include low bit-error rate, interference rejection, and selective addressing capability [19]. This way a much higher signal-to-noise ratio (SNR) can be achieved for the same measurement. The spread spectrum method uses a noise-like signal such as a pseudorandom binary sequence (PRBS) to spread the input signal in the frequency domain, thus, making use of the full bandwidth of the communication channel and reducing the instantaneous power of the signal. For TOF measurements the input signal is a delta function in time, and thus it is the PRBS signal itself that is transmitted. By applying the spread spectrum approach to single photon counting, the response recorded by the TCSPC card (G^SS^(t)) does not resemble a conventional TOF histogram. Instead, the response is the convolution of the transmitted PRBS with the system’s IRF and the impulse response of the medium, i.e.:

(3) $$G^{\text{SS}}(t)={\text{IRF}}^{\text{SS}}(t)*P(t)*{\text{TPSF}}_{\text{Real}}(t),$$

with *P*(t) denotes the binary sequence with which the optical transceiver is modulated. In order to apply the spread spectrum technique to our setup, a maximum-length sequence (MLS) was chosen to be optically transmitted, due to its excellent autocorrelation properties, which are similar to a delta function [20]. MLSs are spectrally flat and provide the maximum possible period if N_MLS_=2^q^-1 for a given degree q. The circular autocorrelation of an MLS is a Kronecker delta function with DC offset and time delay, depending on selected implementation. For a zero-symmetric mapping, its autocorrelation (*R_XX_*) is given by:

(4) $$R_{XX}(\tau )=\frac{1}{T}\int_{0}^{T}P(t)P^{*}(t-\tau )dt=\{\begin{array}{l} 1,\;\text{for}\;\tau /T=0\\ -\frac{1}{N_{\text{MLS}}},\;\text{otherwsise} \end{array}$$

where P^*^ denotes the complex conjugate, *τ* is the time delay and T is the transmitted sequence period. By cross-correlating the TCSPC card response, G^SS^(t), with the binary MLS *P*(*t*) we have:

(5) $${\text{TPSF}}^{\text{SS}}(\tau )=〈G^{\text{SS}}(t)P(t-\tau )〉={\text{IRF}}^{\text{SS}}(\tau )*R_{XX}(\tau )*{\text{TPSF}}_{\text{Real}}(\tau ),$$

with TPSF^SS^(*τ*) representing the measured TPSF of the subject under test, valid only when the TPSF’s duration is less than the transmitted MLS period.

So far, other research groups have demonstrated the benefits of spread spectrum techniques into TD NIR imaging by using either bulky PRBS generators [21–25] or FPGAs [26], programmed to generate PRBSs to modulate fast vertical-cavity surface-emitting lasers (VCSELs), as also shown in [27], where an FPGA was employed, in order to generate a dual-channel 2^10^-1 PRBS at 2.5Gb/s line rate. The core principle in [27] relies upon the modulation and demodulation of the produced PRBSs with a low-frequency reference signal, by means of an analogue modulator (AM), thermoelectrically cooled avalanche photodiodes and a data acquisition (DAQ) device, which eventually leads to a significant compromise with respect to the resolution of the system’s IRF and acquired TPSFs.

### 2.2. System configuration

**Fig. 1:**
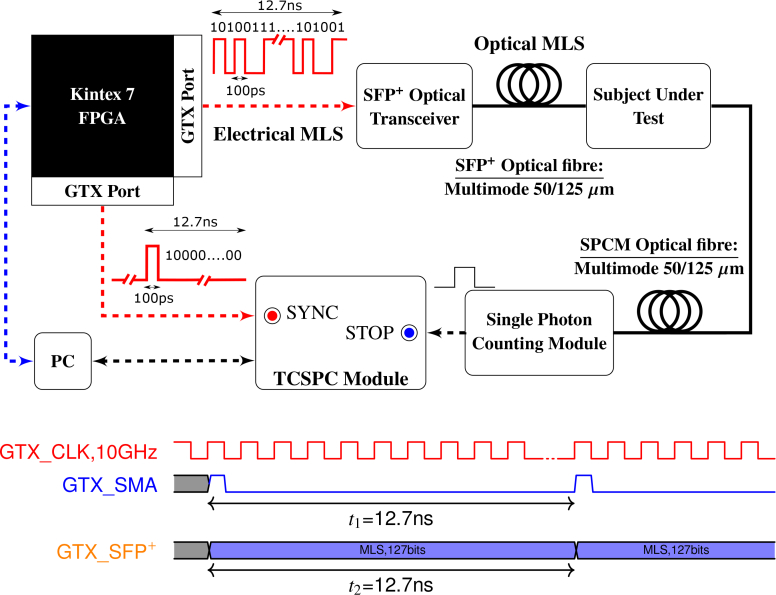
A schematic representation of the implemented TD NIRS system. At the bottom of the Figure, a simplified timing diagram demonstrating the relationship between the operating clock of the GTX transmitters and the outputs of the GTX SMA and the GTX SFP^+^ ports.

A schematic diagram of the proof-of-concept, developed instrument for TD NIRS can be found in Fig. 1

. The system exploits the use of a Gigabit Optical transceiver (AFBR-709SMZ, Avago), utilising an 850nm VCSEL, a TCSPC card (DPC-230, Becker & Hickl), a single photon counting module (SPCM) using a thermoelectrically cooled and temperature controlled silicon avalanche photodiode (SPCM-AQR-14-FC, PerkinElmer) and the Multi-Gigabit Transceivers (MGTs) of a Kintex 7 FPGA (KC705, Xilinx). MGTs are in practice embedded Serialise/De-serialise (Ser-Des) devices, providing built-in solutions for gigabit bandwidth applications that require the transmission or reception of data using the FPGA [28]. The MGTs of Kintex 7 are called *GTX Transceivers* and are used as a basic block for common interface protocols (e.g. PCIe and SATA) [29]. The Kintex 7 FPGA features sixteen GTX ports, which can drive similar Gigabit optical transceiver modules in parallel, and modulate them in a synchronous or asynchronous manner, supporting line speeds from 500Mb/s to 12.5Gb/s. The selected optical transceiver is part of a family of enhanced small form-factor pluggable (SFP^+^) modules, supporting data rates up to 16Gb/s. The transceiver’s VCSEL is Class 1 with 0.79mW maximum output power, which is eye safe under all circumstances. The radiant power of the accessible laser beam is always below or equal to the maximum permissible exposure value, according to the BS EN 60825-1:2014 British standard. The module’s overall power dissipation does not exceed 1W (typ. 600mW).

As Fig. 1 illustrates, in the proposed setup two GTX ports of Kintex 7 FPGA were employed, both programmed to operate at 10Gb/s in a synchronous manner, clocked by the same, dedicated GTX reference clock. A fixed length MLS is transmitted from the SFP^+^ port via the optical transceiver to the sample with fixed period. Similarly, a single 100ps width electrical pulse is sent to an SMA output port with the same period. The fast electrical pulse stemming from the SMA output of the FPGA board is used as the **SYNC** electrical signal for the TCSPC acquisition board. Single photon detection is performed by the SPCM and a single-ended TTL pulse is produced for every detected photon. The TTL signal from the SPCM acts as the **STOP** electrical signal for the TCSPC acquisition board. Accuracy in the various timings of the GTX process is of paramount importance and is ensured by employing an on-board ultra low jitter (<0.32ps) crystal-to-low-voltage differential signalling (LVDS) clock generator as our system’s GTX reference clock (ICS844021I, IDT). Both source and detector are fibre-coupled to the samples under test by means of multimode glass optical fibres with 50/125 *μ*m of core/cladding. The electrical pulses are transmitted to the TCSPC card through standard high-frequency SMA/BNC cables. In order to obtain the correct polarity and amplitude for the optimal TCSPC card operation, the electrical pulses also pass through pulse inverter (A-PPI-D, Becker & Hickl) and appropriate attenuation modules.

**Fig. 2:**
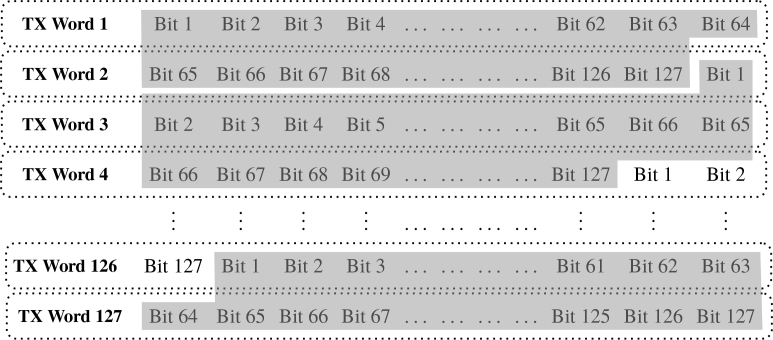
A simplified representation of the internal architecture of the ROM file loaded into the FPGA, in order to generate 127-bit sequences, i.e. pulses and MLSs. With this structure a 127-bit MLS and a single pulse were generated, both having a 12.7ns period. In dark grey the full 127-bit transmitted sequences are highlighted, split into two or three TX words.

The GTX transceiver can be configured to transmit data of any of the following available widths, i.e. 16, 20, 32, 40, 64 or 80 bits. Taking into consideration normal TPSF times (typically around 4–6ns) and the fact that transmission needs to be performed with 10Gb/s line rate, an MLS with q=7, i.e. 127-bit width was chosen, corresponding to a sequence repetition rate of 78.74MHz. The transmitted MLS was first generated in Matlab (Mathworks, Inc.) using a linear-feedback shift register algorithm which was automatically transferred to a read-only memory (ROM) file that was subsequently loaded into the FPGA. Because a 127-bit MLS was too long to be transmitted at once, the MLS was split into 64 bit segments into the ROM file as shown in Fig. 2

, transmitted in a circular manner. This method ensures that MLS data will always be transmitted without any transmission data loss or delay. It is worth mentioning that an MLS generator can be easily implemented on the FPGA, *feeding* its output data into the GTX without the need to use a ROM file.

### 2.3. Collected data post-processing

**Fig. 3:**
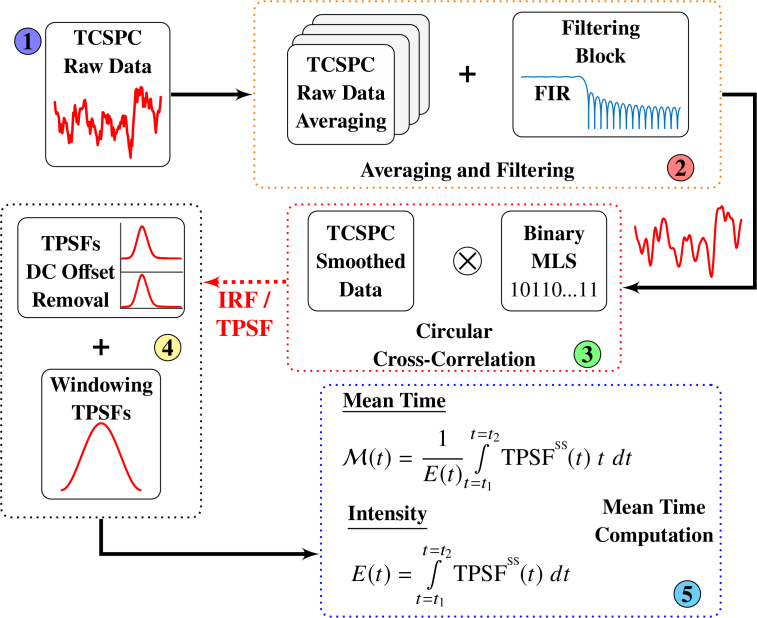
Flow diagram of the five post-processing stages been followed after the collection of the raw MLS data from the TCSPC acquisition board.

An indicative example of a typical TCSPC card response after the MLS excitation can be seen in the *TCSPC Raw Data* block of Fig. 3

. The data are sent automatically to the user’s PC, where the post-processing steps shown in Fig. 3 take place.

The post-processing stages presented in Fig. 3 are standard procedures for TCSPC data post-processing, apart from the cross-correlation step in stage 3, arising from the *unconventional* excitation method. More specifically, raw data are acquired with a fixed integration period and stored in multiple files. Subsequently, at stage 2, averaging and filtering of the raw data takes place. The digital filter employed is a standard low-pass finite impulse response (FIR) with N_FIR_=30 and cut-off frequency at 2GHz. At stage 3 the TCSPC raw data are cross-correlated with the optically transmitted 127-bit long binary MLS. The product of this cross-correlation is the sought after 127 points IRF or TPSF. Stage 4 introduces some additional processing, by subtracting the DC offset from the produced IRF and TPSFs and applying a time window. Finally, stage 5 is where mean time is calculated from the measured data.

## 3. Characterisation of the proposed system

A set of standardised experiments was performed, in order to characterise various aspects of the proposed system, evaluating both its hardware and software performance. The system was characterised across a range of performance metrics, closely following the *Basic Instrumental Performance* protocol proposed by [30], including responsivity, differential non-linearity (DNL) of the timing electronics and IRF’s full width at half maximum (FWHM). Moreover, long term stability, linearity, accuracy and noise were investigated. In Table 1 the properties of the VCSEL source are summarised.

**Table 1:** Properties of the VCSEL light source.

Laser OMA output power (mW)	0.37 (min.)
Laser mean output power (mW)	0.79 (max.)
*λ* (nm)	850
Δ*λ* (nm)	10
Sequence repetition rate (MHz)	Programmable (in this work 78.74)
Time to Initialise (ms)	300 (max.)

**Fig. 4:**
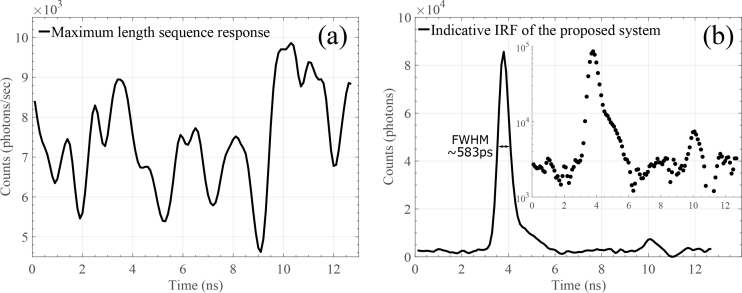
(a) An indicative MLS response as recorded by the TCSPC card using an IRF setup and (b) the resulting IRF after cross-correlation (in linear- and log-scale).

The responsivity of the detection channel is ∼1.55×10^−8^ m^2^sr and the DNL of the timing electronics of the system was found to be <1%, implying good uniformity of the width of the time channels. The system’s IRF FWHM was found to be ∼583ps, as shown in Fig. 4

. Figure 4(a) demonstrates an indicative response of the TCSPC acquisition system, once the aforementioned MLS is optically transmitted by the SFP^+^ module. Figure 4b reveals the IRF, once the MLS response of Fig. 4(a) is cross-correlated with the transmitted binary MLS (stage 3 of Fig. 3). In Fig. 4(b) a distinct second peak appears, relatively long after the primary peak (∼10ns). This peak is likely to be due to a reflection occurring during the IRF measurement. From the inset of Fig. 4(b) the dynamic range of the IRF can be seen, which is one or two orders of magnitude lower than other reported conventional state-of-the-art TD NIRS instruments [11,12]. This seems to be an inherited property of the proposed source, once a spread spectrum technique is applied, that is difficult to overcome with commercially-available components. However, as it can be also seen in the various experimental results below, the system’s overall performance is acceptable for the proposed applications.

### 3.1. Stability

**Fig. 5:**
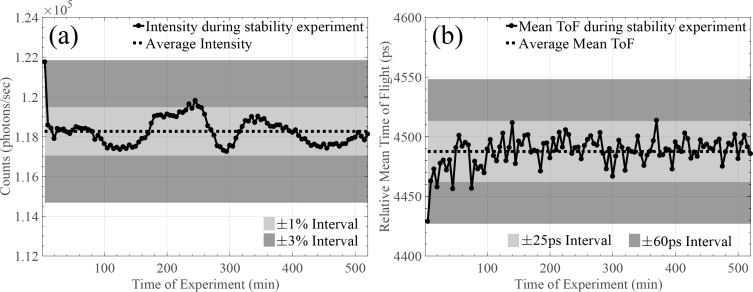
Stability experiment for (a) the intensity and (b) the photon relative mean TOF, using the proposed system over eight hours.

To investigate the stability of the system we recorded the system’s intensity and relative mean TOF variations over an eight hour period. The results, summarised in Fig. 5

, indicate intensity and relative mean TOF stability during the whole experiment. Following an initial *warm-up* period of thirty minutes, the instrument demonstrated intensity stability of ±1% corresponding to standard deviation of ∼715 photons and stability in mean TOF of ±25ps. During the thirty minute stabilisation period, intensity drops and relative mean TOF increases slightly, however, the maximum intensity change does not exceed 3%, while for the relative mean TOF the maximum change does not exceed 60ps. Compared to the aforementioned conventional state-of-the-art TD NIRS instruments [11,12], our system is able to stabilise significantly faster, achieving similar stability performance and decreasing *warm-up* time by a factor of 10 (30min instead of the reported 300min in [12]).

### 3.2. Linearity and accuracy

For the evaluation of the system’s linearity and accuracy, homogeneous liquid-based phantoms were employed, in which suitable absorbing and scattering agents were added to deionised water to control the absorption and reduced scattering coefficients (*μ*_a_ and $\mu_{s}^{\prime }$ respectively) of the final solution. To accommodate the liquid-based phantoms, a custom-made clear acrylic tank was fabricated with dimensions 120×120×35mm. NIR absorbing dye S109564 (ICI, U.K.) was selected to adjust the absorption coefficient, while 20% W/V intralipid (Fresenius Kabi, U.K.) was used to modify the reduced scattering coefficient. For the characterisation of the near-infrared absorbing dye, a 1×1cm cuvette was inserted into a NIR spectrometer (PerkinElmer, USA) to measure the transmittance between 650nm and 950nm. From this spectrum the Beer-Lambert law was used to calculate the dye’s absorption coefficient. The reduced scattering coefficient of the intralipid between 650nm and 950nm was obtained using a broadband TD-NIRS instrument in our group [31]. With knowledge of their spectral properties, we were able to quantify the exact concentration of each component required in a specific volume of deionised water to achieve the desired absorption and reduced scattering coefficients in our phantoms.

**Fig. 6:**
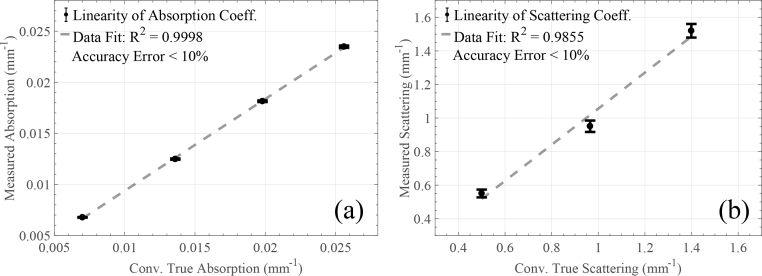
Linearity for the (a) absorption and (b) reduced scattering coefficients at 850nm. The error bars demonstrate the standard deviation between repeated measurements (10 for each point) and the dashed lines represent the first order linear fit of their mean values.

Two sets of experiments were conducted. To determine the linearity of the recovered absorption coefficient, the scattering coefficient was held constant at $\mu_{s}^{\prime }$ = 0.8mm^−1^, whilst the absorption coefficient was varied over the range 0.007 ≤ *μ*_a_ ≤ 0.026mm^−1^. For the second experiment, where the linearity for reduced scattering coefficients was measured, *μ*_a_ value was held constant and set equal to 0.01mm^−1^, while $\mu_{s}^{\prime }$ was increased to achieve nominal values of 0.5, 0.965 and 1.4mm^−1^. Ten measurements on each phantom were taken. The experimental TPSFs were subsequently fitted to a standard model of photon diffusion theory [32] by using a nonlinear least-squares fit algorithm in Matlab. Before fitting, the ideal TPSFs produced by the model were convolved with our system’s IRF. The linearity and accuracy results for absorption and reduced scattering are summarised in Fig. 6

. In both Fig. 6(a) and Fig. 6(b), the dashed lines are the first order linear fit of the measured points, with coefficient of determination for the absorption coefficients R^2^=0.9998 and R^2^=0.9855 for the reduced scattering coefficients. In both cases, the relative accuracy error for each measurement ranged between 2 and 9%.

### 3.3. Noise

The Gigabit optical transceiver achieves fast modulation speeds by exploiting an internal *optical DC offset*, where light is modulated above and below this level. This inherited property of the optical transceiver combined with the applied spread spectrum technique, which *spreads* the system’s noise across the whole frequency spectrum uniformly, results into a TD NIRS system that does not follow traditional noise statistics. More specifically, we expect the noise in our system to be constant and Gaussian, unlike the Poisson type of noise that exists in conventional TD NIRS systems. As one would expect for a Gaussian statistics, noise can be averaged away by increasing integration time. In order to validate the above, the following set of experiments was performed.

**Fig. 7:**
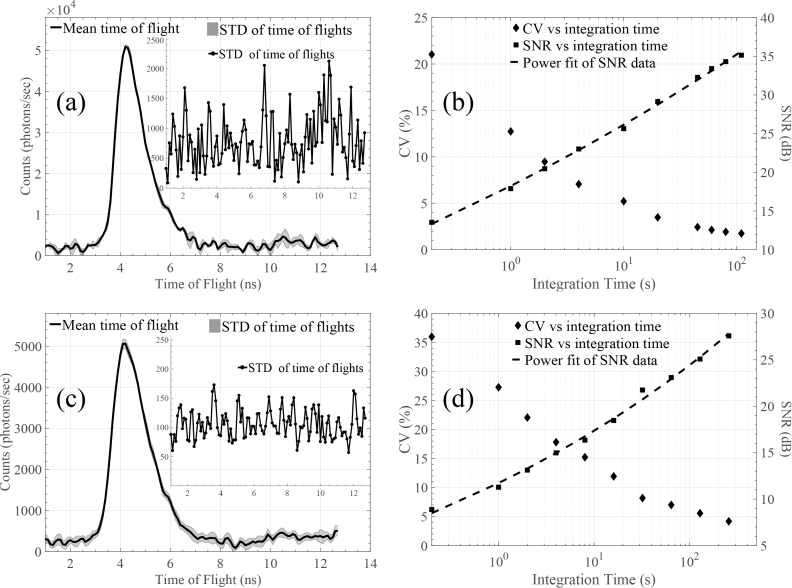
Noise properties of the proposed system. (a) Indicative TPSF from slab with ∼10^6^ counts/sec. (b) The relationship between CV and SNR with integration time for the ∼10^6^ counts/sec case. (c) Indicative TPSF from slab with ∼10^5^ counts/sec. (d) The relationship between CV and SNR with integration time for the ∼10^5^ counts/sec case.

The developed proof-of-concept system does not have yet the ability to control the output power of the laser source and consequently the overall intensity. Therefore, in order to change count rate, different turbid media were employed. Two count rate cases were selected for our noise experiment. In the first one, a thin slab was chosen in transmittance geometry, resulting in a count rate of about 10^6^ photons/sec. For the second case, a much thicker slab was selected in reflectance geometry, resulting into a count rate of approximately 1.15×10^5^ photons/sec. In both cases, TPSFs were collected with integration time set at 0.2 seconds. In order to investigate the impact of integration time upon the noise levels of the system, the 0.2 seconds TPSFs were averaged (as shown in Fig. 3, stage 2), allowing us to obtain different integration time values. For both experiments, the coefficient of variation (CV) and the system’s SNR were calculated. The results for the first set of experiment can be seen in Fig. 7(a) and Fig. 7(b)

, while for the second one in Fig. 7(c) and Fig. 7(d).

Figure 7(a) demonstrates the mean value and the STD of indicative TPSFs, obtained using the high count rate setup. The integration time here was set equal to 2 seconds. Similarly, in Fig. 7(c) mean value and STD of indicative TPSFs, obtained using the low count rate setup and 20 seconds integration time, are shown. As expected, the STD in both cases is constant due to the spread spectrum technique. Moreover, in both cases, there is a linear relationship between integration time and the system’s SNR. The overall behaviour of the two experimental setup is also consistent. For example, in the first case, in order to obtain a CV equal to 5% and SNR around 25dB, a 10 seconds integration time is required. Similarly in the second case, the aforementioned CV and SNR values are obtained when integration time is roughly 100 seconds.

## 4. Phantom and *in vivo* evaluation experiments

In this section TD-related results are presented using both a tissue-equivalent phantom and also by performing a proof-of-concept *in vivo* experiment.

### 4.1. Evaluation on a tissue-equivalent phantom

In this experiment a tissue-equivalent phantom was employed which was already presented and described in detail in [33]. The phantom consists of a solid block of epoxy resin (dimensions 95×175×60mm) with uniform optical properties and a cylindrical cavity through which a rod (diameter of 10mm and length of 130mm) can be manually translated back and forth. A three-dimensional representation of the phantom can be seen in [33]. Based on the values provided, the rectangular block has a transport scattering coefficient $\mu_{s}^{\prime }$=1.0mm^−1^ and absorption coefficient *μ*_a_ = 0.0112mm^−1^ at 850nm. The rod has exactly the same optical properties with the rectangular block apart from its central *target* region, which has an absorption coefficient *μ*_a_ = 0.112mm^−1^. This means that maximum attenuation should be observed when the target is translated across the centre of the cavity.

**Fig. 8:**
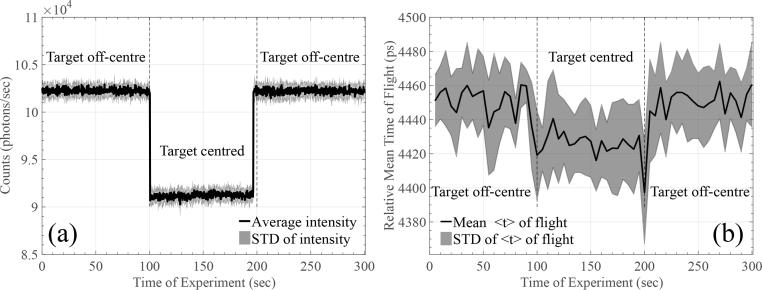
(a) Intensity and (b) relative mean TOF changes during tissue-equivalent rod phantom experiments.

The source and the detector were positioned on the top of the surface with a ∼35mm distance between them, as described in [33]. For this experiment we selected to perform a 300 seconds continuous measurement comprising of three stages: (a) we kept the target *off-centre* for 100 seconds; (b) translated the target across the centre of the cavity for 100 seconds; (c) returned the target back to its *off-centre* position and record for another 100 seconds. Ten measurements were performed and the integration time for each TPSF was set equal to 5 seconds. Figure 8

and Fig. 9

**Fig. 9:**
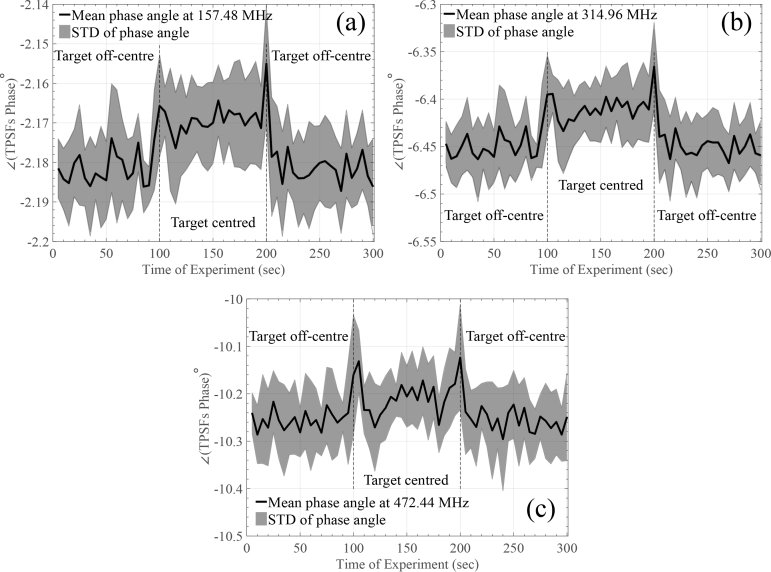
Phase angle changes of the FFT performed on the captured TPSFs for different frequency components for the tissue-equivalent rod phantom experiments.

summarise the obtained results of the experiment.

In Fig. 8(a) the recorded intensity change is shown, related to the target’s position. As approximated in [33], this change (relative to a homogeneous block and the change in the absorption coefficient) corresponds to approximately 0.5dB. Figure 8(b) demonstrates relative mean TOF changes over experiment time. An approximately 20ps–25ps mean TOF change can be seen during the different phases of the experiment. The *spikes* observed at 100 and 200 seconds in Fig. 8(b) are due to momentary exposure to ambient light (the selected SPCM can detect single photons of light over the 400nm to 1060nm wavelength range). Finally, Fig. 9 demonstrates another TD-related information for the phase angle of the Fast Fourier Transform (FFT) performed on the captured TPSFs for different frequency components (bins) of the FFT. It can be seen that frequency bins up to 470MHz reveal the change in the optical properties of the rod phantom. Once again, *spikes* observed at 100 and 200 seconds are due to momentary ambient light exposure. The results of Fig. 9 also verify that the post-processing stages of Fig. 3 did not affect the mean TOF results.

### 4.2. Arterial cuff occlusion in the arm

**Fig. 10:**
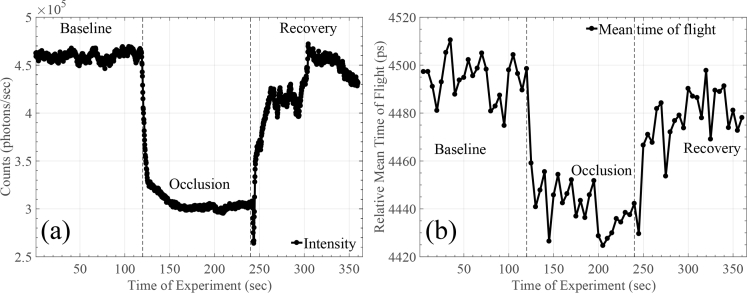
(a) Intensity and (b) relative mean TOF changes during cuff occlusion measurement.

A standard arterial cuff occlusion (200mmHg) of the left arm was performed on an adult male subject. The protocol followed consists of three stages: In the first one the arm was in resting position for 2 minutes; the cuff was rapidly inflated to a pressure of 200mmHg to provide an abrupt vascular (venous and arterial) occlusion, maintained for another 2 minutes; and finally the cuff was released and the recovery phase followed for 2 more minutes. The source and detector were placed on the subject’s forearm 25mm apart and were stabilised with bandages. The experiment was performed in dark room conditions with a blackout material also been placed around the source and detector on the subject’s forearm. Once again, the integration time for each TPSF was set equal to 5 seconds. Figure 10

and Fig. 11

**Fig. 11:**
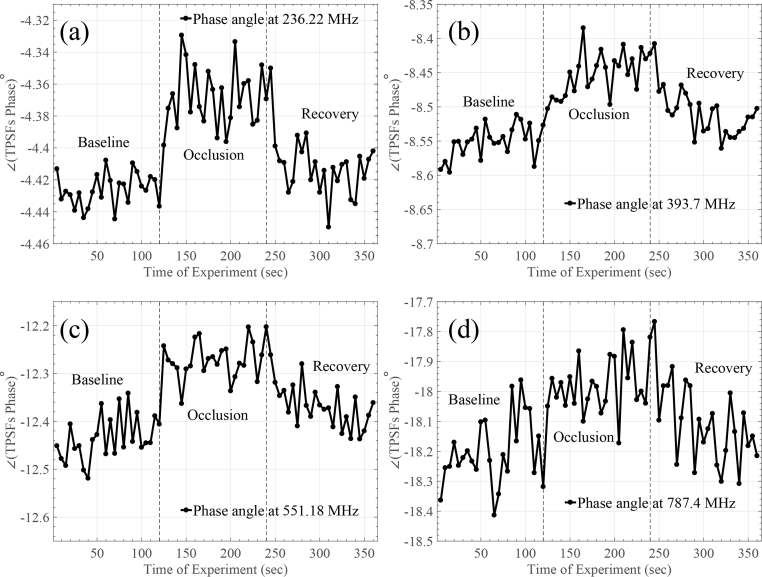
Phase angle changes of the FFT performed on the captured TPSFs for different frequency components for the cuff occlusion measurement.

summarise the obtained results of the experiment.

In Fig. 10, intensity and relative mean TOF changes during the experiment are shown. More specifically, in Fig. 10(a), a steady intensity baseline can be seen for the first 120 seconds. During cuff inflation intensity drops and stabilises around 150 seconds (roughly 30 seconds after onset of occlusion). Subsequently, around 240 seconds, when the cuff is released intensity gradually returns towards its original baseline. The total intensity change corresponds roughly to 1.8dB. Figure 10(b) illustrates the relative mean TOF during the cuff occlusion experiment. It exhibits similar trends to the intensity curve, with a mean TOF change corresponding to ∼60ps. Finally, Fig. 11 demonstrates changes on the phase angle of the FFT performed on the captured TPSFs for four different FFT frequency components (bins). In this experiment, where the change is slightly bigger compared to the previous rod-phantom experiment, it can be seen that frequency bins up to 787MHz can reveal the change in the optical properties of the tissue. Once again, the results of Fig. 11 verify that the post-processing stages of Fig. 3 did not affect the mean TOF results.

## 5. Discussion

The characterisation results in Section 3 demonstrated clearly the pros and cons of the aforementioned system/technique for TOF resolved measurements. More specifically, the responsivity and DNL of the proposed system is comparable with the state-of-the art systems shown in [15]. Our system’s IRF is approximately 1.5–3 times wider compared to other published TD NIRS instruments. However, as literature also indicates, for (f)NIRS applications such system IRF is acceptable, since it is well within the sub-ns range. The dynamic range of the IRF is one or two orders of magnitude lower than other reported conventional state-of-the-art TD NIRS instruments [15]. As already mentioned previously, the limitation in dynamic range seems to be a combination of the optical transceiver’s modulation capabilities (sacrifices dynamic range for speed) and the spread spectrum approach, which *spreads* the system’s noise across the whole frequency spectrum uniformly, thus, deviating from the Poisson noise of conventional TD NIRS systems. The Gigabit optical transceiver achieves fast modulation speeds by exploiting an *optical DC state*, where light is modulated above and below this state. This means that compared to traditional instruments, the baseline of our TPSFs will be noisier. On the other hand, stability and linearity/accuracy is comparable to traditional systems. As shown in Fig. 5, our system is stable within the first ∼30 minutes, achieving ±1% intensity and ±25ps mean TOF temporal stability, a time period significantly lower compared to conventional reported systems that require an order of magnitude more time to reach similar intensity and mean TOF values. The linearity for both absorption and reduced scattering is satisfying, given the R^2^ of Fig. 6, and accuracy error ranged between 2 and 9%. The cost (∼£60) and size (47.5×14×13mm) of the laser source combined with the reported performance makes the system an attractive candidate for portable, low-cost solutions to similar applications.

The evaluation results of Section 4 indicate that we are able to extract meaningful TD-related information. The tissue-equivalent rod phantom experiment shows that the system is capable of detecting small intensity changes, in the order of 0.5dB, and small mean TOF changes around 20–25ps with integration time set equal to 5 seconds. Longer integration times (e.g. 10 seconds) provide more distinct mean TOF changes, however, we appreciate that these values might not be very practical for challenging functional experiments. The results of the rod-phantom experiment demonstrate that a function task detection should be possible with the same integration period and reasonable averaging. Finally, our proof-of-concept *in vivo* experiment shows much larger intensity and mean TOF changes, due to the bigger change in optical properties of the subject under test. Both evaluation experiments are accompanied by useful phase angle change graphs. For the rod phantom case, frequency bins up to 470MHz can reliably reveal the change in the optical properties of the subject, while in the *in vivo* experiment frequency bins up to 787MHz can be used to extract information from the sample.

By using, to the best of our knowledge for the first time in NIRS, mature telecommunication transceiver modules, a single channel proof-of-concept system was developed, which exhibits not only performance comparable with conventional pulsed TD NIRS systems but also versatility. The small footprint, low-cost optical transceivers modulated by the FPGA at high line rates can transmit various types of optical sequences with sequence repetition rates that can be easily defined by the user, ranging from few MHz up to hundreds of MHz, depending on the application. The large number of MGT ports existing in standard modern FPGAs allow for the simultaneous modulation of many optical modules with different patterns in a synchronous or asynchronous manner. Switching time between the multiple optical modules can take place almost instantaneously, overcoming the slow switching problems that exist in some conventional TD NIRS instruments.

Work is ongoing to improve the presented system even further. One of the most important improvements would be to enable spectroscopic measurements, therefore, future work will concentrate on the development of custom-made SFP^+^ optical transceiver modules, where VCSELs of different wavelengths will be driven by standard IC drivers, allowing us to control the speed and output power of the optical transceiver. Whilst the *transmit side* of the electronics is nicely integrated, the *receive side* still uses multiple pieces of hardware which could be integrated into the FPGA fabric, reducing significantly the system’s overall size and cost. More specifically, the TCSPC module can at later stages be substituted by a custom-made, picosecond range time-to-digital converter, implemented entirely on the FPGA platform [34,35], reducing the complexity and the size of the total experimental setup even more (in practice only the FPGA platform and a SPCM will be needed). Finally, the relatively high-cost SPCM could be substituted later by silicon photomultipliers, which have been developed in recent years as an alternative to traditional photomultiplier tubes, allowing the whole setup to be even smaller, without compromising speed or accuracy. The width of our system’s IRF is mainly due to the selected SPCM, whose timing properties and limitations are reported at length in [36]. An alternative choice of detector, and the use of graded index optical fibres could allow a substantial reduction in the FWHM of the IRF.

## 6. Conclusion

We have developed and characterised a proof-of-concept, single channel TD NIRS system, relying upon the spread spectrum technique. The proposed system utilises for the first time in literature a commercially available, low-cost, optical transceiver module, widely used in telecommunication applications, as a light source controlled by a Kintex 7 FPGA, which modulates the optical transceiver with MLS at 10Gb/s. The preliminary characterisation results of the system as well as the encouraging preliminary tissue-equivalent phantom and *in vivo* evaluation results demonstrate the potentials of this instrument as an alternative to conventional TD NIRS instruments, once more channels with different wavelengths are included. The specific approach to TD NIRS is still in its infancy, however, the obtained proof-of-concept results combined with the low-cost and small footprint of the instrument allow us to proceed even further with this new TOF resolved technique, not only for biomedical but also for industrial purposes.
